# Aerial Parts of *Peucedanum chenur* Have Anti-Cancer Properties through the Induction of Apoptosis and Inhibition of Invasion in Human Colorectal Cancer Cells

**DOI:** 10.29252/ibj.24.5.309

**Published:** 2020-03-04

**Authors:** Saeed Yadegari, Massuod Saidijam, Mohammadreza Moradi, Dara Dastan, Ali Mahdavinezhad

**Affiliations:** 1 Research Center for Molecular Medicine and Genetics, Hamadan University of Medical Sciences, Hamadan, Iran;; 2 Medicinal Plants and Natural Products Research Center, Hamadan University of Medical Sciences, Hamadan, Iran;; 3 Department of Pharmacognosy and Pharmaceutical Biotechnology, School of Pharmacy, Hamadan University of Medical Sciences, Hamadan, Iran

**Keywords:** Antioxidant, Apoptosis, Colorectal neoplasm, Medicinal plant, Neoplasm invasiveness

## Abstract

**Background:**

The *Peucedanum* species have many pharmacological effects due to the presence of coumarins, flavonoids, phenolic compounds, and essential fatty acids in these species. In this study, for the first time, the anticancer activity of *Peucedanum chenur* methanolic extract via the induction of apoptosis and inhibition of invasion in HCT-116 human colon cancer cells was investigated.

**Methods:**

*P. chenur* methanolic extract effect on HCT-116 cells viability and antioxidant activity were evaluated using MTT assay, DPPH, and iron chelating tests, respectively. Changes in mRNA expression level in a panel of relevant genes were assessed by the quantitative real-time PCR. Also, apoptosis was assessed by cell cycle analysis and Annexin V/PI method, and the effect on cell migration was tested using scratch test.

**Results:**

*P. chenur* methanolic extract increased significantly the expression of *BAX* while decreased the expression of *BCL-2*, *AKT1*, *FAK*, *RhoA*, and *MMP* genes compared to the control group. *BAX/BCL-2* ratio and apoptosis elevated, whereas cell migration reduced significantly. Besides, our extract showed an appropriate antioxidant activity.

**Conclusion:**

*P. chenur *may be introduced as a new chemopreventive agent in medicine due to its notable power in terms of induction of apoptosis and inhibition of invasion.

## INTRODUCTION

Colorectal cancer is the third common cancer in the world^[^^1 ▶^^]^. Current methods for treating CRC include surgery, chemotherapy, and radiation therapy^[^^2 ▶^^]^. Resistance to apoptosis and tissue invasion are key features of cancer cells^[^^3 ▶^^]^. On the other hand, cell migration, as a complex process, critically contributes to cancer invasion and metastasis^[^^4 ▶^^]^. Cancer progression occurs due to changes in the cell microenvironment and activation of extracellular proteases, thereby causing tumor cell motility and moving to distant tissues through the bloodstream, which results in metastasis^[^^5 ▶^^]^.

Nowadays, herbal medicinal products have gained more attention than synthetic drugs because they are cheaper, safer, and more accessible and have fewer drug interactions^[^^2 ▶^^,^^6 ▶^^]^, and unlike synthetic compounds, they influence several signaling pathways in cancer cells^[^^7 ▶^^]^. Studies have shown that some natural compounds can induce apoptosis in cancer cells, which leads to homeostasis in tissues^[^^8 ▶^^,^^9 ▶^^]^. Therefore, finding a suitable natural compound to induce apoptosis in cancerous cells and inhibit invasion activity of them is considered as a scientific achievement. 

The genus *Peusedanum* has more than 120 species and grows in Europe, Asia, and Africa. The pharmacological properties of *Peucedanum *species are attributed to coumarin, flavonoids, phenolic compounds, and essential fatty acids^[^^10 ▶^^]^. *Peucedanum chenur* is an endemic species of Kurdistan Province in the west of Iran, and to the best of our knowledge, the anticancer properties of this species has not yet been assessed. Therefore, in this study, the anticancer effects of* P. chenur* methanolic extract on HCT-116 human colon carcinoma cells were investigated. Further to general assays like MTT assay and scratch test, we tried to determine the expression levels of apoptosis and invasion related genes such as *BAX*, *BCL-2*, *AKT1*, *FAK*, *RhoA*, and *MMP-13*. Also, antioxidant activity of the plant extract was evaluated by two relevant assays,DPPH and iron ions chelating. This survey is the first study aimed at investigating the mechanism of action for antitoxicity effect of the *P. chenur*.

## MATERIALS AND METHODS

**Preparation of** ***P. chenur*** **extract**

*P. chenur* was collected from Kurdistan Province, Iran. A voucher specimen (no. 2951) was registered in the herbarium of the Research Institute of Forests and Rangelands, Sanandaj, Iran. Aerial parts of *P. chenur* (180 g) were cut into small pieces and an Erlenmeyer flask to extract methanolic extract according to maceration method through shaking. After 72 hours, methanolic extract was passed through a Whatman filter paper and concentrated in vacuum at 50 °C using a rotary evaporator. The extract was kept in a sterile vial in the dark and a cool place until use. 

**Cell culture**

HCT-116 and Vero cells were prepared from the National Cell Bank of Iran, Pasteur Institute of Iran, Tehran. The cells were cultured in a T25 flask using DMEM medium (Thermo Fisher Scientific, USA) containing 10% FBS (Thermo Fisher Scientific), 100 U/ml of penicillin, and 100 µ/ml of streptomycin (Kiazist Life Sciences, Iran). Incubation conditions included a temperature of 37 C, 5% carbon dioxide, and 95% relative humidity. 

**Cell viability assay**

Viability of extract-treated HCT-116 and Vero cells was investigated using the MTT assay in a dose -and time-dependent manner in the same way. Optimum cell density was determined according to the method used by Moradi *et al.*^[^^11 ▶^^]^, and 6 × 10^3^ cells were seeded in each well of a 96-well plate. The cells were kept at 37 C for 24 hours, then different concentrations of methanolic extract of *P. chenur* (20, 60, 100, 140, 180, 220, 260, 300, 340, and 380 µg/ml) were added to each well. The first and second rows were selected as the negative and positive controls (by adding 21 µl/well of DMSO in order to see apoptotic form of HCT-116 cells), respectively. After incubation times (24, 48, and 72 hours), 10 μl (5 mg/ml) of MTT solution (Sigma Aldrich, USA) was added to each well and was incubated again at 37 °C for 4 hours. Subsequently, in a low light environment, entire contents of the wells were removed, and 100 μl of DMSO was added to each well to dissolve formazan crystals. Finally, OD was measured by an ELISA reader at 570 nm, and experiments were repeated three times. To find optimum IC_50_ within 24, 48, and 72 hours, Pearson correlation coefficient was calculated to show correlation between the viability of cells and concentrations. IC_50_ value for 48-hour incubation time (182.1 µg/ ml) was considered as optimum concentration for the next experiments. To determine IC_50_ value for Vero cells, the cells were treated with final concentrations of 0, 360, 420, 480, 540, and 600 µg/ml of extract in each well for 48-hour incubation time.

**Gene expression analysis**

***RNA extraction and cDNA synthesis***

As mentioned earlier in cell viability assay Section, IC_50_ value (182.1 µg/ml) for 48-hour incubation time was considered as optimum concentration for the next experiments. Therefore, incubation time of methanolic extract of *P. chenur* for real-time PCR test was 48 hour. Total RNA of the treated cells was extracted by RNX-plus (CinnaGen, Iran) according to the instruction of the kit. Quality and concentration of the extracted RNA were assessed by 2% agarose gel electrophoresis and Nanodrop (Biotech, USA). cDNA synthesis was performed using the Primecript RT reagent kit (Takara, Japan) as per the manufacturer’s instruction. 

***Real*** ***-*** ***time PCR***

Gene expression analysis was performed using quantitative real-time PCR technique by Roche LightCycler® 96 system according to Moradi *et al.*’s method^[^^12 ▶^^]^. Specificity of primer pairs was investigated by NCBI primer blast, and quantitative real-time PCR was performed in duplicate on mixture containing l μl of 8 pmol/mL specific primer pairs (Table 1), 7 μl of water, 10 μl of SYBR Premix Ex Taq II (Takara), and l μ1 of cDNA. *GAPDH* gene was used to normalize the data, and relative expression analysis was performed using 2^-ΔΔCT^ method. Products were confirmed through 2% agarose gel electrophoresis.

**Table 1:** Characteristics and sequence of primers

**Gene name**	**Accession number**	**Primer sequence**
*GAPDH*	NM-002046.6	Forward: 5′-AAGGCTGTGGGCAAGGTCATC-3′ Reverse: 5′-GCGTCAAAGGTGGAGGAGTGG-3′
*BAX*	NM-138761.3	Forward: 5′-CGCCGTGGACACAGACTC-3′ Reverse: 5′-GCCTTGAGCACCAGTTTG-3′
*BCL-2*	NM-000657.2	Forward: 5-TGGAGAGTGCTGAAGATTGA-3 Reverse: 5-GTCTACTTCCTCTGTGATGTTGTAT-3
*AKT1*	NM-005163.2	Forward: 5′-GTGGCTATTGTGAAGAGA-3′ Reverse: 5′-GGATGATGAAGGTGTTGG-3′
*FAK*	NM_001352696.1	Forward: 5'-CCTCGCAGTCATTTATCATCAG-3' Reverse: 5'-CTCCAATACATCGTCCAAGTTC-3'
*RhoA*	NM_001313946.1	Forward: 5'-ATAGTGGATGAGCTGTGAGTGC-3' Reverse: 5'-ACCAGACCGTGGACTAACGA-3'
*MMP-13*	NM_002427.3	Forward: 5'-AGTTCGGCCACTCCTTAGGT-3' Reverse: 5'-TGGTAATGGCATCAAGGGAT-3'

**Sub-G1 DNA content assays**

To reveal the effect of *P. chenur* on apoptosis, HCT-116 cells were exposed to various concentrations of the extract (0, 140, 180, and 220μg/ml) for 48 h. Briefly, 200 μl of PI (10 μl of PI, 0.5 μl of RNase, and 189.5 μl of PBS buffer) was added to the prepared sample and incubated at 37 °C until injection to the flow cytometry instrument. Ratio of Dead (Sub-G1 phase) cells was calculated by a flow cytometry device (Partec, Germany).

**Annexin V/PI assay**

Annexin V-FITC/PI staining (eBioscience, USA) was performed to confirm apoptosis. At first, 4 × 10^6^ cells were seeded in four flasks and incubated at 37 °C. After 24 hours, the culture medium was disposed, and the cells were incubated for 48 h in different concentrations of *P. chenur* extract (140, 182.1, and 220 μg/ml). After 48 hours, the cells were collected, and 1 ml of PBS was added to the cell precipitate and then centrifuged at 1000 ×g , 4 °C for 5 minutes. The supernatant was discharged, and depending on the precipitate concentration, 300 to 350 μl of reagent buffer was added to the cellular deposition and was incubated for 15 minutes. Next, Annexin V/PI staining was conducted to detect apoptotic and necrotic cells, which was as follows: 5 μl of Annexin V was added to the suspension and was incubated for 30 min at ambient temperature. Then 5 μl of PI was augmented, followed by 15-min incubation time at ambient temperature. Finally, suspensions were injected in a flow cytometry device (Becton-Dickinson, San Diego, CA, USA) and analyzed by CellQuest software Version 5.2.

**Scratch test**

Scratch test is an easy and a reliable way to investigate the effect of chemical substances on the ability of cell proliferation and migration in *in vitro* studies. First, 75 × 10^3^ cells were seeded per each well of a 24-well plate in DMEM high glucose medium. After 24 hours, when cell confluence reached 80–90%, scratches were made in each well by a sterile 100-μl pipette tip, and debris was removed through washing with PBS. The cells were incubated for 48 h in the diverse concentrations of *P. chenur* extract (140, 182.1, and 220 μg/ml). Then the images were taken from four groups, at time 0 and 48 hours later, in three independent experiments, and wound closure was measured by the NIH ImageJ software in treated and control groups. In order to improve the accuracy of measurement of relative migration, the captured images were changed into binary images according to the method used by Khodadadi *et al.*^[^^13 ▶^^]^.

**Antioxidant assay**

Antioxidant activity of the extract was evaluated by DPPH and iron ions chelating assays^[^^14 ▶^^]^. Antioxidant activity of different concentrations of *P. chenur* extract was calculated as a percentage relative to the positive control. IC_50_ value was defined as an effective concentration of our extract needed for scavenging 50% of radical activity and chelating 50% of iron ions in DPPH and iron chelating assay, respectively^[^^15 ▶^^]^.

***DPPH radical scavenging***

DPPH is a chemical dark powder compound with the molecular weight of 394.32 g/mol and is used to determine the amount of free radicals in the system. This compound is radically soluble in ethanol and has the maximum absorbance at 517 nm. It becomes more stable by taking electrons, and its absorption decreases at 517 nm. Briefly, 200 μl of different concentrations of *P. chenur* extract and BHA were prepared in a similar way (in 95% of methanol). Methanol and BHA were used as the negative and positive controls, at the same order. Then 800 μl of DPPH solution (0.15 mM), was dissolved in 95% ethanol and was added to each sample. Finally, all samples were mixed well and were incubated in the dark at room temperature for 30 minutes. Absorbance of all solutions was measured at 517 nm. The following equation shows how antioxidant activity of the extract was calculated^[^^16 ▶^^]^, where, A_control_ and A_sample_ are the amounts of control and sample absorbance at 517 nm, respectively.

([A_control_–A_sample_]/A_control_) × 100 = DPPH free radical scavenging (%)

***Iron chelating activity***

By applying iron-chelating test, we measured the percentage of iron ions absorbed by a substance^[15]^. After adding iron dichloride (FeCl_2_) to a solution containing *P. chenur* extract, the extract compounds began to absorb iron ions and did not allow the reaction between free iron ions and ferrozine molecules. Ferrozine, with a molecular weight of 492.46 g/mol, acts as an indicator for iron ions in the solution^[^^17 ▶^^]^. At first, 200 μl of different concentrationsof the extract and EDTA were prepared in methanol, and 200 μl of methanol without extract and EDTA were used as the controls. Then 500 μl of distilled water was added to each microtube, followed by vigorous spin. Next, 100 μl of FeCl2 was added and was incubated for 3 minutes at ambient temperature. In the final step, 200 μl of 5 mmol of Ferrozine was added and was incubated in dark at room temperature for 10 minutes. Absorbance of all solutions was measured at 562 nm. The iron chelating effect was calculated according to the following equation, where A_control_ and A_sample_ are the amounts of control and sample absorbance at 517 nm, respectively^[^^17 ▶^^]^.

Iron chelating activity (%) = [(1-(A_Sample_/A_Control_)] × 100

**Statistical analysis**

Data were analyzed using GraphPad Prism 6 (Graph Pad Prism Inc., USA). Quantitative variables were presented as mean ± standard deviation (mean ± SD). Differences between the groups were analyzed by one-way ANOVA and *t*-test. The *p *< 0.05 was considered as statistically significant.

## RESULTS

**Effect of** ***P. chenur*** **methanolic** **extract** **on cell viability**

**Fig. 1:**
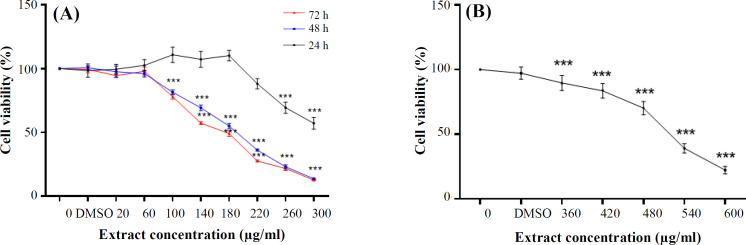
Toxicity effect of *P. chenur* methanolic extract on HCT-116 and Vero cells. (a) HCT-116 cells were treated with various concentrations of extract, followed by MTT assay. The extract reduced the vitality of the cells compared to the control group. (b) Vero cells, which were treated with different concentrations of the *P. chenur *extract showed cytotoxic effects at concentrations >IC_50_ value for HCT-116 cells (360 µg/ml). Data are presented as the mean ± SD (n = 8); ^***^ *p* ˂ 0.001

In this study, the effect of different concentrations of extract on HCT-116 cells was investigated using MTT assay. As it is demonstrated in Figure1A, our extract reduced the percentage of viable cells compared to the control group, in a dose- and time-dependent manner with an IC_50_ value of approximately 182.1 µg/ml and 163.2 µg/ml after 48 and 72 hours of incubation, respectively. Also, Vero cells, as normal cells treated with different concentrations of *P. chenur* extract, showed no significant decrease in the cell viability 

**Fig. 2:**
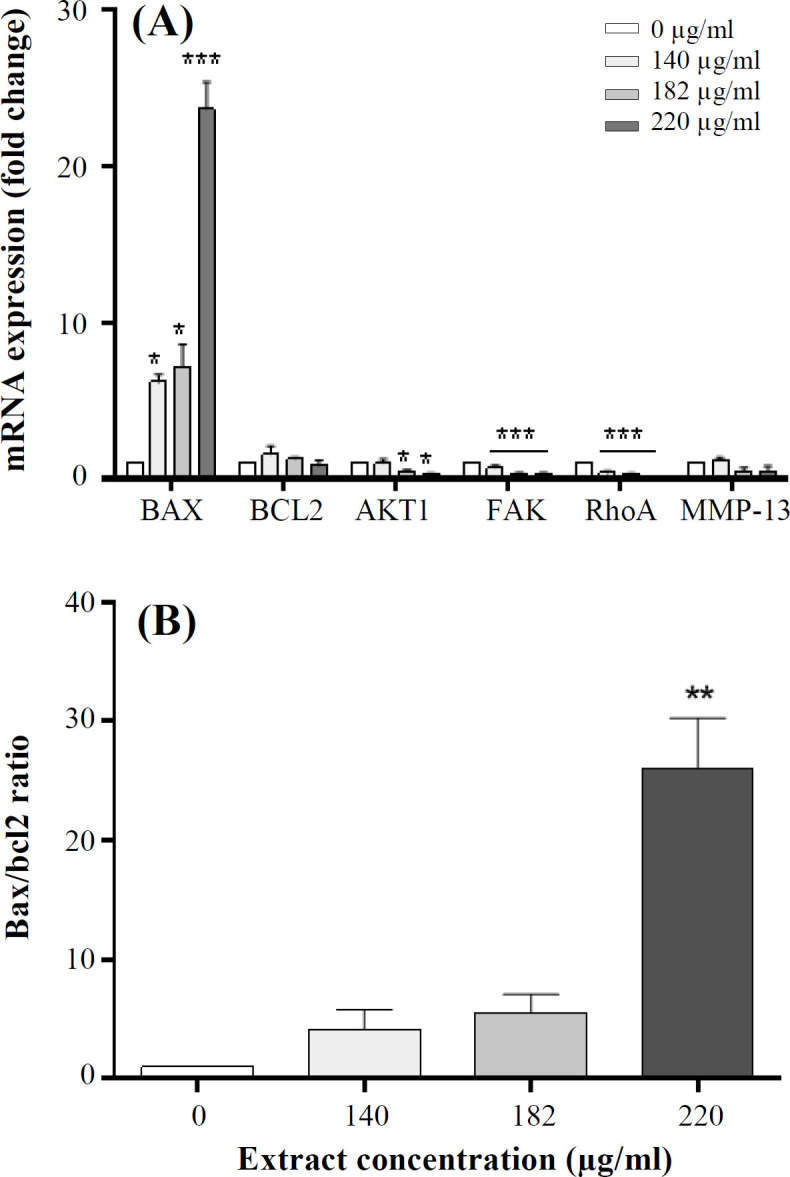
Gene expression effects of *P. chenur* methanolic extract (A) Effect of the extract on expression of *BAX*, *BCL-2*, *AKT1*, *FAK*, *RhoA*, and *MMP-13* genes in HCT-116 cells within 48 hours. (B) Increased *BAX/BCL-2* ratio observed in real-time PCR resulted in extract-treated cells vs. -untreated cells. Data are presented as the mean ± SD. ^*^p < 0.05, ^**^p < 0.01, ^***^p < 0.001 vs. the control

even up to a concentration of 360 µg/ml (Fig. 1B). IC_50_ value for Vero cells within 48 hours was equal to 517.9 μg/ml. 

**Effect of** ***P. chenur*** **methanolic** **extract** **on the expression of** ***BAX*** **,** ***BCL-2*** **,** ***AKT1*** **,** ***FAK*** **,** ***RhoA*** **, and** ***MMP-13*** **genes**

As shown in Fig. 2A, *P. chenur* extract increased the expression of *BAX* gene as well as decreased *FAK*, *RhoA*, and *MMP-13* genes significantly in the treated groups compared to the control group. Also, the expression of BCL-2 decreased at the concentration of 220 μg/ml of our extract, but it was not significant. Similarly, a significant decrease was found in the expression of AKT1 at the concentrations of 182 μg/ml and 220 μg/ml. *BAX/BCL-2* ratio elevated significantly compared to the control group (Fig. 2B).

**Sub-G1 DNA content and apoptosis measurement**

**Fig. 3:**
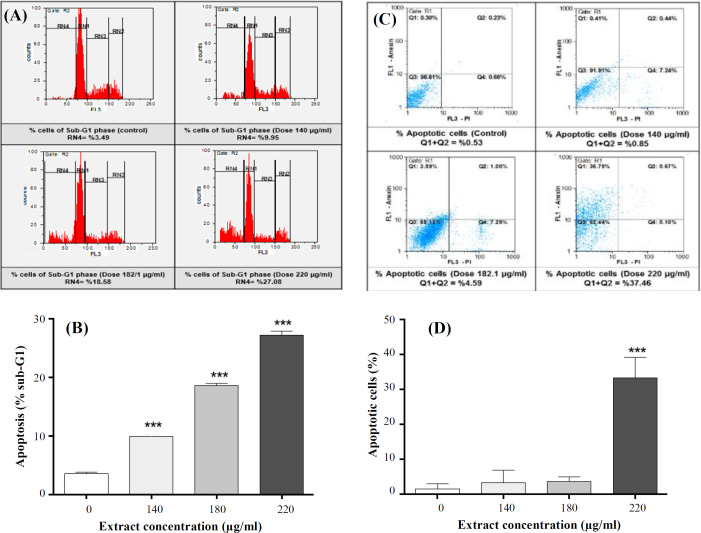
Effect of *P. chenur* on cell cycle progression and apoptosis**. (A) ***P. chenur* methanolic extract effect on cell cycle alteration. (B) The fraction of Sub-G1 cells increased in the treated groups in a dose-dependent manner. (C) The effect of the extract on the induction of apoptosis in HCT-116 cells. (D) Apoptotic cells increased in treated groups in a dose-dependent manner compared to the control group. Data are presented as the mean ± SD (n = 3. ^*** ^*p ˂ *0.001).

In order to evaluate the effect of *P. chenur* on cell distribution in different phases of cell cycle, flow cytometric analysis was carried out in cells treated with various concentrations of *P. chenur* methanolic extract. Results demonstrated that the extract increased the percentage of cellular accumulation in the Sub-G1 phase in treated groups compared to the control group in a dose-dependent manner (Fig. 3A and 3B and Table 2). For the confirmation of the apoptotic effect of *P. chenur*, the cells were stained with Annexin V/PI. A significant increase was observed in the percentage of AnnexinV/PI positive cells in cells treated with *P. chenur* methanolic extract compared to the control group (Fig. 3C and 3D and Table 3).

**Effect of** ***P. chenur*** **methanolic** **extract** **on antioxidant activity (DPPH free radical scavenging** **and iron ions chelating)**

**Fig. 4:**
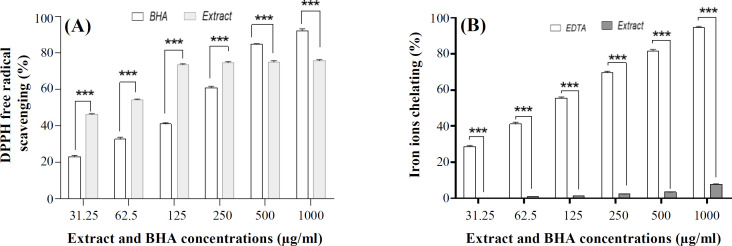
The antioxidant effect of *P. chenur* methanolic extract. (A) DPPH free radicals scavenging at various concentrations of extract compared to the similar concentration of standard antioxidant (BHA). (B) Percentage of iron ions chelating at different concentrations of *P. chenur* extract compared to the same concentrations of EDTA. Results are presented as the mean ± SD (n = 3). ^***^p < 0.001

 Results showed that *P. chenur* has higher ability to scavenge DPPH radicals than BHA, as a strong synthetic antioxidant. DPPH free radical scavenging at the concentrations of 182.1 μg/ml of methanolic extract of *P. chenur* and BHA was equal to 63.61% and 45.17%, respectively. Also, IC_50_ values (for DPPH free radical scavenging) of extract and BHA were equal to 46.17 μg/ml and 251.7 μg/ml, at the same order. Furthermore, it was found that *P. chenur* methanolic extract has iron ions chelating property. Iron ions chelating activity at the concentrations of 182.1 μg/ml of *P. chenur* extract and EDTA was equal to 1.39% and 53.12%, respectively. IC_50_ values of our extract and EDTA were equal to 6.34 μg/ml and 130.41 μg/ml, correspondingly. Based on the results, a direct relationship was observed between *P. chenur* methanolic extract concentration and DPPH free radical scavenging as well as iron ions chelating activity (Fig. 4). 

**Migration assay**

**Fig. 5:**
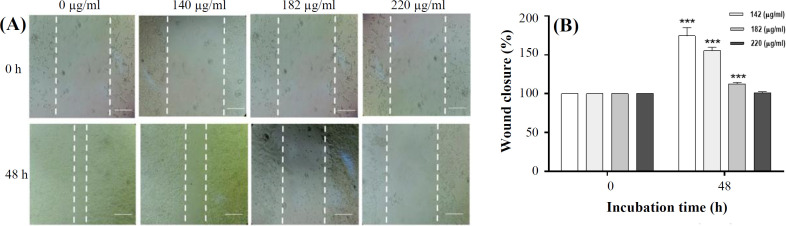
*P. chenur* methanolic extract suppressed cell migration. (A) Representative image of the extract effect on migration of HCT-116 cells treated with different concentrations of *P. chenur* at time zero and 48 hours later. Migration rate of the cells reduced in the treated groups compared to the control group in a dose-dependent manner. (B) Wound closure was quantified randomly in three fields in all groups. Data are presented as the mean ± SD (n = 3). ^***^p < 0.001 vs. control

Scratch test was used to measure the effect of methanolic extract of *P. chenur* on cell migration. As shown in Figure 5, the number of migrated cells and wound closure in all the treated groups decreased significantly compared to the control group so that the migration rate was inhibited perfectly at the concentration of 220 μg/ml of *P. chenur*.

## DISCUSSION

CRC is the second leading cause of cancer-related death in the world^[^^18 ▶^^]^. There is no proper procedure for early detection of CRC, and common cancer treatments have many side effects^[^^19 ▶^^]^. Over the past 20 years, more than 50% of commonly used drugs have been derived directly from plant derivatives, or some of the plant derivatives were considered as patterns to prepare some effective drugs^[^^2 ▶^^]^. The effects of *Peucedanum *to chemopreventive activity^[^^20 ▶^^]^, anti-proliferative activity^[^^21 ▶^^,^^22 ▶^^]^, induction of apoptosis^[^^23 ▶^^]^, growth retardation^[^^24 ▶^^]^, antioxidant properties^[^^14 ▶^^]^, limitation of cellular invasion^[^^25 ▶^^]^, and synergistic effects with other anticancer drugs^[^^22 ▶^^]^. 

 species on cancer cells can be attributed 

For the first time, the results of our study showed anti-proliferative and suppressive ability of *P. chenur* methanolic extract on human colon cancer HCT-116 cells, while under the same condition, it did not inhibit cell proliferation of Vero cells (IC_50_ value at concentration of 182.1 μg vs. 517.9 μg, respectively). To the best of our knowledge, anticancer effects of limited species of this plant, including *Peucedanum nebrodense*^[^^21 ▶^^]^ and *Peucedanum japonicum*^[^^26 ▶^^]^, have been investigated. For instance, the effect of *P. japonicum* extract on induced colon cancer in F344 rats has been reported^[^^20 ▶^^]^. 

Our survey on apoptosis-related genes revealed that *P. chenur* methanolic extract up-regulates *BAX* gene significantly in treated groups, but it down-regulates the expression of *BCL-2* and *AKT1*. Besides, *BAX/BCL-2* ratio increased. Inducible apoptosis and cell cycle alteration in HCT-116 cells by *P. chenur* were supported by flow cytometric analysis. Considering our results, it could be proposed that the apoptotic effects of *P. chenur* methanolic extract occurs through the mitochondrial pathway. 

Cancer development is strongly associated with impaired apoptosis process^[^^27 ▶^^]^ and immortal cancer cells show a major alteration in apoptosis pathways^[^^28 ▶^^]^. In mitochondrial-dependent apoptosis pathway, *BAX* (pro-apoptotic) proteins create an external channel in mitochondrial membrane, thereby entering cytochrome C to cytoplasm and connecting to Apaf-1 and procaspase-9 complexes and then forming apoptosome complex^[^^9 ▶^^]^. Apoptosome activates porocaspase-9 proteolysis, and activated *caspase-9* is released, which activates executive Caspase cascades such as Caspase-3^[^^29 ▶^^]^. In the absence of apoptosis stimulator, *BCL-2* is activated and prevents all of these events and causes cell survival^[^^30 ▶^^]^. Also, phosphorylation of Caspase-9 is induced by active *AKT1* protein which, in turn, decreases the activity of *Caspase-9* and inhibits cellular apoptosis^[^^31 ▶^^]^. Moreover, decline of *BAX/BCL-2* ratio results in resistance to apoptosis^[^^32 ▶^^]^, and reduced expression ratio of *BAX/BCL-2* genes in colon cancer causes apoptosis resistance^[^^32 ▶^^]^.

**Table 2:** Results of *P. chenur* effect on cell cycle alteration

**Concentration** **(** **µg/ml)**	**Sub-G1** **(%)**	**G1** **(%)**	**S** **(%)**	**G2/M** **(%)**
control	3.49 ± 0.05	60.71 ± 0.06	20.21 ± 0.03	13.78 ± 0.04
140	9.95 ± 0.01	54.01 ± 0.05	19.50 ± 0.03	16.11 ± 0.02
182.1	18.58 ± 0.2	55.33 ± 0.5	15.00 ± 0.3	12.13 ± 0.2
220	27.08 ± 0.6	47.25 ± 0.9	11.63 ± 0.2	13.26 ± 0.3

Data are presented as the mean ± SD (n = 3).

Our reasults were confirmed by the study conducted by Fong *et al.*^[^^23 ▶^^]^ who reported that pyranocoumarin purified from *Peucedanum praeruptorum* extract increases *Bax* and *BAX/BCL-2 *ratio in HL-60 leukemia cells. They also indicated that the mentioned extract causes apoptotic DNA, nucleolar fragnmentation, and induction of apoptosis in multidrug-resistant cancer cell lines^[^^23 ▶^^,^^33 ▶^^]^. 

Migration is a complex process that plays an important role in the invasion and metastasis of cancer cells^[^^4 ▶^^]^. Active *AKT1* increases invasion intensively^[^^4 ▶^^]^. *FAK*, as a tyrosine kinase, is associated with signaling between cells and extracellular matrix and acts as a scaffold protein in cellular connections in metastatic colon cancer and overexpresses in metastatic colon cancer^[^^34 ▶^^]^. *RhoA* is a small *Ras*-family GTPase that contributes to the organization of cellular skeletons, invasion, transcription, and cell proliferation. It also controls the formation of focal cell adhesion and stress-bearing strands in the cell^[^^35 ▶^^]^. Overexpression of *RhoA* has been proved in colon cancer^[^^36 ▶^^]^. 

*MMPs* are proteolytic enzymes that break down the extracellular matrix and cause cellular migration and metastasis. Expression of *MMP-13*, as a member of collagenases in the cancerous tissue, is significantly higher than healthy tissue^[^^37 ▶^^]^. *FAK*, by activating RhoA, regulates the structure of cellular skeleton and cell-cell communication^[^^38 ▶^^]^ and adjusts the contraction force of migration by regulating *RhoA* and *MMPs* in separate pathways^[^^39 ▶^^]^. *MMP-13 *has a very important function in tumor invasion and metastasis in most malignancies by degrading type II collagen. Compared to breast or lung cancer cell lines, *MMP13* is highly expressed in CRC cell lines. *MMP13* mRNA up-regulation is correlated with tumor size, tumor invasiveness, and lymph node metastasis^[^^40 ▶^^,^^41 ▶^^]^.

In the present study, *P. chenur* methanolic extract reduced the expression of *AKT1*, *FAK*, *RhoA*, and *MMP-13* genes in HCT-116 cells. Furthermore, a significant decrease in cell migration was confirmed by the scratch test in HCT-116 cells treated with the methanolic extract of *P. chenur*, even migration was inhibited completely in IC_50_ value at the concentration of 220 μg. In agreement with our research, *P. japonicum* ethanol extract has been found to inhibit invasion by decreasing *MMP-9* expression and inhibits *PKC*/*Nf-KB* signaling pathway in MCF-7 breast cancer cells^[^^25 ▶^^]^. These results suggest the inhibitory effect of our extract on motility and migration of tumoral cells through *FAK*/*Rho*-*A/MMP-13* signaling pathways; thus, it can be proposed as a new chemopreventive agent to prevent metastasis.

Oxidation is a vital process in living cells. Free radicals are one of the oxidation products and are highly reactive and harmful molecules influencing the vital macromolecules and aggravate progression of the cancer^[^^42 ▶^^]^. Antioxidant substances can protect the cell from oxidative damage through neutralizing the activity of free radicals^[^^43 ▶^^]^. Antioxidant effect of several genus of *Peucedanum* has been confirmed^[^^14 ▶^^]^. 

**Table 3:** Results of AnnexinV/PI assay

**Concentration** **(** **µg/ml)**	**Necrotic cells** **(%)**	**Late apoptotic** **cells (%)**	**Early apoptotic** **cells (%)**	**Viable** **cells (%)**
control	7.98 ± 2.0	1.4 ± 1.3	0.13 ± 0.1	90.49 ± 1.1
140	7.73 ± 0.7	1.57 ± 1.6	1.77 ± 1.9	88.93 ± 4.2
182.1	7.81 ± 0.7	1.44 ± 0.6	2.18 ± 1.98	88.57 ± 0.6
220	1.17 ± 1.5	1.89 ± 1.7	31.33 ± 7.7	65.61 ± 4.5

Data are presented as the mean ± SD (n = 3).

The methanolic extract of *Peucedanum graveolens* has a high thermal and pH stability, and its antioxidant effect increases at high temperatures and pH levels^[^^44 ▶^^]^. In this study, two antioxidant tests, DPPH and iron chelating, were used^[^^14 ▶^^]^. Results clearly indicated a direct association between antioxidant activity and *P. chenur* methanolic extract concentration. In DPPH antioxidant test, higher antioxidant activity of our extract was shown compared to standard antioxidants, BHA (63.61% vs. 45.17%), as well as lower IC_50_ value to scavenging free radicals (46.17 μg vs.251.7 μg).

Although iron chelating test showed that our extract has lower ability to chelate iron ions compared to EDTA as a standard compound, IC_50_ value for the methanolic extract of *P. chenur* and EDTA were estimated to be about 6.34 μg and 130.41 μg, respectively. Tepe *et al.*^[^^45 ▶^^]^ suggested that essential oils obtained from *Peucedanum longifolium* and *Peucedanum palimbioides* extracts have 8.59% and 10.67% free radical scavenging activity of DPPH at 0.2 mg of concentration and have 12.42% and 90.39% Iron ions chelating activity at 2 mg of concentration, respectively. Meanwhile, DPPH free radicals scavenging IC_50_ for polyphenols and flavonoids derived from *Peucedanum Pastinacifolium* hydroalcoholic extract has been indicated to be equal to 469.4 μg, and this value for iron chelating was equal to 657.5 μg^[^^14 ▶^^]^. As a result, *P. chenur* methanolic extract has higher antioxidant ability compared to other *Peucedanum* species at lower concentration; thus, *P. chenur* methanolic extract may be introduced as a new antioxidant agent in the field of medicine in the future.

Altogether, our study, for the first time, showed the notable anti-proliferative effect of *P. chenur *methanolic extract in the induction of apoptosis through mitochondrial pathway as was evident from the increased *BAX/BCL-2* ratio and the inhibition of invasion through the reduction of *AKt1*, *FAK*, *RhoA*, and *MMP-13* expression. It also may be proposed as a strong anti-oxidative agent in the future studies, due to its ability to remove free radicals. Collectively, this natural agent may be turn out as a novel therapeutic strategy in colon cancer treatment.
